# Interpolated Retrieval of Relevant Material, Not Irrelevant Material, Enhances New Learning of a Video Lecture In-Person and Online

**DOI:** 10.3390/bs15050668

**Published:** 2025-05-14

**Authors:** Zohara Assadipour, Dahwi Ahn, Jason C. K. Chan

**Affiliations:** 1 Department of Psychology, Iowa State University, Ames, IA 50011, USA; ckchan@iastate.edu; 2 Department of Psychology, University of Waterloo, Waterloo, ON N2L 3G1, Canada; d3ahn@uwaterloo.ca

**Keywords:** learning, memory, retrieval practice, forward testing effect, context change, lecture, strategy change account, engagement, test-potentiated new learning, interpolated retrieval

## Abstract

Interpolated retrieval enhances the learning of new information—a finding known as the forward testing effect. The context change account suggests that learning benefits are due to a shift in internal context, which can be triggered through the retrieval of either content-relevant or content-irrelevant information. In two experiments, we examined whether interpolated episodic, autobiographical, and semantic retrieval would enhance new learning of a video lecture, compared to interpolated review. Participants watched a STEM topic lecture divided into three ~5 min segments and completed their assigned interpolated activity after the first two segments. Across both a laboratory (Experiment 1, *N* = 249) and online setting (Experiment 2, *N* = 246), only episodic retrieval enhanced the learning of new material; autobiographical and semantic retrieval (content-irrelevant) did not improve new learning. Critically, we introduced a measure of context change to determine whether the level of engagement in these interpolated activities predicted recall. Engagement correlated with criterial test performance when controlling for effort (seriousness). Our results support a multi-factor explanation for the forward testing effect, providing evidence for both the context change and strategy change accounts, although we emphasize that support for context change should be interpreted with caution.

## 1. Introduction

Students often need to learn and retain substantial amounts of information, particularly in academic settings where college lectures frequently exceed 40 min. Problematically, research has consistently shown that students’ ability to learn decline over the course of extended learning sessions (Johnstone & Percival, 1976; Risko et al., 2012), and this effect is particularly pronounced in online classes (Ahn & Chan, 2022; Francis et al., 2019). To address this challenge, interpolated retrieval practice (IRP)—whereby a lesson session is broken up into smaller segments by having students answer questions about what they have learned between the segments—has been proposed as an effective intervention (Brame, 2016; Hew, 2018). Specifically, IRP enhances the retention of previously studied material (Murphy et al., 2023; Roediger & Karpicke, 2006; Rowland, 2014). But more importantly for present purposes, it also facilitates the learning of new information, a phenomenon known as the forward testing effect (FTE) or test-potentiated new learning (Chan et al., 2018b, 2020, 2025; Pastötter & Bäuml, 2014; Yang et al., 2018).

Evidence of the forward testing effect (FTE) was first reported by Tulving and Watkins (1974) using word-pairs in an A-B, A-C paired-associate learning paradigm. Specifically, participants learned two lists of pairs that shared the same cues, and the key manipulation of this study was whether or not participants completed an interpolated test for the first list (A-B) before they studied the second (A-C). In a criterial test (i.e., a final assessment of learning) that required participants to recall all targets associated with a cue (both B and C in response to A), participants recalled more of the first list (B) words than the second list (C) words if they completed an interpolated drawing task from their short-term memory. However, this effect was eliminated when participants took an interpolated test between the lists, because the interpolated test promoted the learning of items in the second list. Subsequent research has shown that engaging in interpolated retrieval practice (IRP) can insulate against the buildup of proactive interference (PI)—a negative effect of prior learning on new learning (Ahn & Chan, 2022; Pastötter et al., 2011; Szpunar et al., 2008)—and enhance list discrimination (Chan & McDermott, 2007a; Jang & Huber, 2008; Pastötter & Bäuml, 2014; Pastötter et al., 2011; Szpunar et al., 2008; Weinstein et al., 2014).

Several theoretical accounts have been proposed to explain this forward testing effect. The context change account postulates that retrieval induces an internal context change when one switches from encoding to retrieval (Davis et al., 2017; Tulving et al., 1994). For example, when participants study items in a list, those items are associated with a List-1 context. According to this account, retrieval of the List-1 items causes a change in internal context, so when participants learn new items afterwards, those items are associated with a different List-2 context. In contrast, when participants do not perform retrieval practice between the two lists, all of the list items are hypothesized to be associated with a single study-phase context. The contextual segregation between Lists 1 and 2 is believed to reduce the detrimental effects of PI and enhance the retrieval of list-specific items (Jang & Huber, 2008; Pastötter et al., 2011; Szpunar et al., 2008), thereby contributing to the occurrence of the FTE.

In addition to episodic retrieval practice, other tasks that differ substantially from episodic encoding are also believed to induce context change. These tasks include performing an N-back task (Pastötter et al., 2011), recalling details of one’s childhood home, or imagining a distinctive scenario such as becoming invisible (Delaney et al., 2010; Jonker et al., 2013; Sahakyan et al., 2013; Sahakyan & Kelley, 2002). Another task that has been suggested to induce context change from episodic encoding is to perform semantic generation, in which participants produce exemplars (e.g., chair, table) based on a category cue (e.g., furniture). A commonality across the aforementioned tasks is that they involved some type of retrieval activity, even if the retrieval targets (e.g., one’s childhood home or exemplars of a category) were irrelevant to the learning task. For example, the semantic generation task requires retrieval from semantic memory, the N-back task requires retrieval from working memory, and the childhood home task requires retrieval from autobiographical memory. At first glance, the imagination tasks described above do not seem to involve retrieval, but tasks that require rich imaginative constructions of scenes often engage brain regions that are critical to episodic retrieval (Schacter et al., 2017; Szpunar et al., 2007, 2009). Of particular relevance to the present study is that inserting these learning-irrelevant retrieval tasks into a multi-list learning paradigm has sometimes produced the FTE (Divis & Benjamin, 2014; Jang & Huber, 2008; Kliegl & Bäuml, 2021; Kriechbaum & Bäuml, 2024; Pastötter et al., 2008, 2011), just like a learning-relevant episodic retrieval task would. Together, these studies provided evidence for the context change account of the FTE.

However, support for the context change account is not universal. For example, Weinstein et al. (2015) did not observe an FTE using interpolated autobiographical retrieval and semantic generation, despite attempting to directly replicate Pastötter et al. (2011), but without the EEG component. Recently, Ahn and Chan (2022) observed that interpolated episodic recall potentiated new learning to a similar degree regardless of whether participants studied inter-related (e.g., both Lists 1 and 2 contained words related to fruits and animals) or categorically distinct word lists (e.g., List 1 contained only fruit words and List 2 contained only animal words). If interpolated retrieval enhances new learning due to context change, the FTE should be much weaker or absent for categorically distinct lists. Because switching categories triggers a context change (Brown, 1958; Peterson & Peterson, 1959; Wickens et al., 1963), it should render the benefits of interpolated retrieval redundant. Therefore, the finding that the FTE was undiminished with the categorized lists relative to inter-related lists posed a problem for the context change account. Likewise, Yang et al. (2019) argued that the context change account would not predict an FTE when participants learn different types of materials across lists (e.g., List 1 contains Swahili–English pairs, and List 2 contains face–name pairs). Yet, interpolated retrieval produced similarly powerful benefits on new learning regardless of whether participants studied the same or different types of material across lists (see also Hong et al., 2019, for evidence inconsistent with the context account for the testing effect).

Some theorists have also questioned the viability of the context change account from a conceptual perspective. One concern is that the extent to which internal context change has occurred is difficult to ascertain. This issue is highlighted by prior work suggesting that interpolated activities like mental arithmetic or counting backwards by three do not induce context change (Divis & Benjamin, 2014; Kliegl & Bäuml, 2021; Pastötter et al., 2011). However, given that these interpolated activities are dissimilar to episodic encoding and are content-irrelevant to the encoded material, one might expect them to induce context change. The assumption that these tasks do not induce context change raises the following question: what makes those tasks differ from semantic generation, an N-back task, or imagining one’s childhood home in terms of context change? Without an independent measure of context change, it is difficult to evaluate this claim directly. Instead, researchers have used improvement in memory performance as evidence for context change. This practice is problematic because it leads to circularity, which has been a long-standing concern for some context change explanations (Riccio et al., 1984, 1999). Context change is a *proposed explanation* for why the FTE happens, so one cannot, in turn, use the occurrence of the FTE to justify context change. In the present study, we aimed to provide a rigorous test of the context change account as an explanation of the FTE, and we introduced a way to quantify context change independently of criterial task performance.

Another prominent explanation for the FTE is the strategy change account, according to which, engaging in retrieval practice promotes future learning because it encourages individuals to apply more effective encoding and/or retrieval strategies later (Chan et al., 2018a, 2018b; Davis & Chan, 2015, 2023). Specifically, taking a test gives the learner metacognitive insights by revealing the test structure, retrieval cue availability, gaps in knowledge, etc., which can inform future learning (Bjork & Storm, 2011; deWinstanley & Bjork, 2004; Hays et al., 2013). For example, after taking a test, participants might be underwhelmed by their performance and attempt to exert a greater effort into learning subsequent material, as demonstrated by increased study time (Davis & Chan, 2023; Soderstrom & Bjork, 2014; Yang et al., 2017). Alternatively, during an interpolated test, participants might learn that the relational properties of studied items can serve as powerful retrieval cues. This realization might cause participants to focus on these properties during subsequent encoding and retrieval opportunities. This type of strategy change can manifest via greater organization in participants’ recall (Chan et al., 2020, 2018a; Jing et al., 2016; Yang et al., 2022). However, the strategy change perspective cannot readily explain why content-irrelevant retrieval tasks, like semantic generation, would promote new learning. Bäuml and colleagues (Kliegl & Bäuml, 2021, 2023) thus proposed that combining strategy change and context change into a two-factor account can address each of the individual account’s shortcomings. Indeed, as other theorists have also noted (Chan et al., 2018b; Yang et al., 2018), the strategy change and context change accounts are complementary rather than mutually exclusive, so combining them can enhance explanatory power without posing conceptual contradictions.

## 2. The Current Study

There is now a considerable body of literature demonstrating the robust nature of the FTE; however, the vast majority of these studies have employed simplistic materials (e.g., word lists, paired associates) that students do not typically encounter in the physical or virtual classroom (for a review, see Chan et al., 2018b). In the current study, we sought to examine whether content-irrelevant IRP can enhance the learning of lecture materials, which remains the most common form of knowledge delivery in both the classroom and online (Hansch et al., 2015; Seaton et al., 2014). To that end, we examined the impact of different types of IRP on the new learning of a video lecture under both in-person (Experiment 1) and online settings (Experiment 2). The strategy change and context change accounts make distinct predictions regarding the effects of different types of IRP on new learning.

In the present study, participants watched a video lecture split into three segments. Immediately following Segments 1 and 2, participants either studied several *review* slides, performed *episodic retrieval* by answering six quiz questions about the immediately prior segment, performed *autobiographical retrieval* by thinking about their childhood home or a vacation, or performed *semantic retrieval* by generating exemplars from category names.

Both the strategy change and context change accounts would lead one to predict that interpolated episodic retrieval would promote new learning—as shown by an increase in the criterial Segment 3 test performance—relative to an interpolated review. We were less certain about whether interpolated autobiographical retrieval and semantic retrieval would promote new learning. On the one hand, the strategy change account predicts that only episodic retrieval (but not semantic or autobiographical retrieval) should potentiate new learning; on the other hand, the context change account might lead one to predict that all interpolated retrieval tasks would promote new learning.

Prior research has employed autobiographical retrieval and semantic generation as methods to induce context change. However, heretofore, no studies have attempted to measure the extent of context change that participants experienced. Indeed, none have reported participants’ performance during these interpolated tasks (Divis & Benjamin, 2014; Kliegl & Bäuml, 2021, 2023). In the present paper, we measured participants’ engagement and success with the interpolated activities. Assuming that mental context change is a continuous rather than a binary phenomenon, participants who are more engaged with the interpolated tasks should undergo greater context change. To this end, we introduce a measure for both autobiographical retrieval and semantic generation. Engagement with the autobiographical retrieval task was measured via a word count after verification that participants were properly following instructions (e.g., they were recalling their childhood home or a vacation rather than something unrelated), and semantic retrieval engagement was measured by the number of correct exemplars recalled.

If mental context change is a driver of the FTE, then one should expect a positive association between engagement with these tasks and criterial test performance. However, before accepting this hypothesis at face value, it must be weighed against an alternative explanation, namely, that participants who engage more with autobiographical or semantic retrieval may exhibit better memory performance not because of context change, but because they were more engaged with the experiment as a whole. In other words, these participants were simply taking the experiment more seriously. To address this alternative hypothesis, we also asked participants to indicate how seriously they took the experiment using a Likert scale question. We report a hierarchical regression analysis to examine the extent to which interpolated task engagement predicts criterial test performance beyond seriousness. Moreover, we detail limitations regarding the interpretation of these data as a proxy for context change in Section 6.

## 3. Experiment 1: Laboratory Study

### 3.1. Methods

#### 3.1.1. Design and Participants

This study adopted a between-subjects design wherein participants were randomly assigned to one of four interpolated activity conditions: (content-relevant) episodic retrieval, (content-irrelevant) autobiographical retrieval, (content-irrelevant) semantic retrieval, or (content-relevant) review. Target sample size was determined based on Pastötter et al. (2011), who reported a minimal effect size of *d* = 0.70 across all interpolated retrieval tasks (content-relevant episodic retrieval, semantic generation, working memory) relative to the control condition of interpolated review or interpolated distractor. To be conservative, we conducted our power analysis using *d* = 0.49, which is 70% of Pastötter et al.’s effect size. When there are more than two levels in the independent variable, the analysis of variance (ANOVA) approach is commonly used for the power analysis. However, because we were examining against the hypothesis that performance on the Segment 3 criterial test would be equivalent in the three retrieval conditions, we used an independent samples *t*-test approach with an unequal allocation ratio (i.e., retrieval/review = 3:1). This analysis indicated that we would need 178 participants (133 in the retrieval conditions and 45 in the review condition) to achieve 80% power at a 0.05 alpha level.

Participants consisted of 249 students (*M*_age_ = 19.06, *N*_reported_ = 226) from a large midwestern university and were randomly assigned to the four conditions. There were 61 participants in autobiographical retrieval, 62 in semantic retrieval, 63 in episodic retrieval, and 63 in review. The experiment was conducted in the laboratory under the supervision of research assistants. Participants received course credit as compensation. See Table 1 for participant demographics.

#### 3.1.2. Materials and Procedure

Participants watched a ~15 min lecture video, which was divided into three ~5 min segments. The lecture covered topics in either statistics or physics. For stimulus sampling purposes, two versions of each lecture were created featuring either a man or woman instructor. Each lecture displayed the instructor in a head-and-shoulder view at the bottom left of the lecture slides. Details about the construction of the lecture videos can be found in Chan et al. (2025).

Participants were instructed to pay attention to the lecture like they would for an actual class, but they were prohibited from taking notes. Participants were told that a randomization algorithm determined whether they would receive quiz questions or review slides following each lecture segment. However, in actuality, participants engaged in their assigned interpolated activity for three minutes following each of the first two segments. All participants took a criterial test of Segment 3 immediately following its presentation.

In the episodic retrieval condition, participants answered six short-answer questions over the just-presented lecture segment (for Segments 1 and 2). For example, a question from the physics lecture asked, “What type of particles do masers emit?” Questions were presented individually for 30 s without feedback, so the episodic retrieval task lasted a total of three minutes. 

In the review condition, participants were shown six review slides over content presented in the previous lecture segment for Segments 1 and 2, with each shown for 30 s. All review slides had equivalent information to the quiz questions in the episodic retrieval condition, such as “Masers emit microwaves and radio waves.”

Participants in the autobiographical retrieval condition were prompted to recall a specific memory as extensively as possible for three minutes. Specifically, they were asked to recall their childhood home following one segment and their most memorable vacation following another segment, with the order of prompts counterbalanced across participants. Participants were warned, in red and underlined letters, not to include any personally identifiable information during their recall. No participants included identifying information.

In the semantic retrieval condition, participants were instructed to recall as many exemplars as possible for a given category. The categories included four-footed animals, furniture, non-alcoholic beverages, professions, sports and vegetables. Participants were given 60 s per category and were shown a randomly chosen, non-repeated three categories following each of the first two lecture segments. 

Following Segment 3, all participants took a criterial test on this segment. This test consisted of six short-answer questions and was administered in the same manner as the interpolated episodic retrieval task. Before dismissal, all participants answered demographic and data quality control questions.

### 3.2. Results

All analyses were conducted using two-tailed tests with an alpha level of 0.05. When reporting effect sizes, we use Cohen’s *d* for pairwise comparisons and partial eta square η_p_^2^ for other comparisons. All analyses were conducted with JASP Team (2024). We report the results regarding context change based on interpolated task engagement following presentation of the results from both experiments. These analyses were based on the combined data from Experiments 1 and 2 because our individual experiments were not powered to detect the association between interpolated task engagement and test performance. All inferential statistics are reported to three decimal places, and descriptive statistics are reported to two decimal places.

#### Do All Interpolated Retrieval Tasks Boost New Learning?

**Figure 1:**
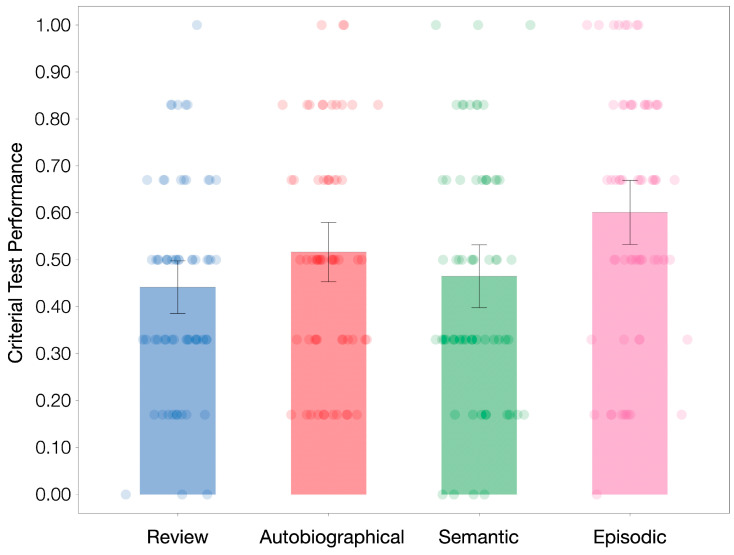
Criterial test performance across conditions of Experiment 1. Dots denote individual participant performance. Error bars denote 95% descriptive confidence interval. Direct comparisons reveal that episodic retrieval, but not semantic retrieval, significantly enhances new learning relative to review. The difference between autobiographical retrieval and review is inconclusive (*p* = 0.078).

The primary question of the present study is to compare the effectiveness of different types of retrieval tasks on new learning. Figure 1 shows the criterial test performance across the four interpolated activities. The context change account posits that retrieval would induce a change in context and reduce proactive interference, so all retrieval conditions should outperform review. An ANOVA was conducted to examine this hypothesis with the independent variable consisting of each interpolated activity condition and the dependent variable as the proportion of correct recall on the criterial test. Results showed a significant main effect of interpolated activity, *F*(3, 245) = 4.96, *p* = 0.002, η_p_^2^ = 0.057. Perhaps most importantly, planned comparisons showed that only interpolated episodic retrieval (*M* = 0.60, *SD* = 0.27) significantly enhanced new learning relative to interpolated review (*M* = 0.44, *SD* = 0.22), *t*(124) = 3.61, *p* < 0.001, *d* = 0.642. Neither autobiographical retrieval (*M* = 0.52, *SD* = 0.25), *t*(122) = 1.78, *p* = 0.078, *d* = 0.319, nor semantic retrieval (*M* = 0.47, *SD* = 0.26), *t*(123) = −0.53, *p* = 0.594, *d* = −0.096, significantly promoted new learning relative to the review. Additionally, lecture topics did not interact with interpolated activity, *F*(3, 241) = 0.97, *p* = 0.410, η_p_^2^ = 0.012. Our results are thus consistent with the strategy change account but not the context change account. Descriptive statistics of each condition are presented in Table 1. One might notice, however, that the effect for autobiographical retrieval was somewhat inconclusive (i.e., its *p*-value was between 0.05 and 0.10). We will address this finding in more detail following presentation of the data in Experiment 2, which will serve as a replication in an online setting.

## 4. Experiment 2: Online Study

### 4.1. Methods

#### 4.1.1. Design and Participants

Experiment 2 was a direct replication of Experiment 1, except that we wanted to generalize the data to online learning. Consequently, all participants completed the experiment fully online. Participants consisted of both students from the same midwestern university as in Experiment 1 (*N* = 49) and those from Prolific (*N* = 200). We began data collection near the end of spring semester, during which university participants were available, but we finished data collection with participants from Prolific after the end of the spring semester. The university students were compensated with course credit, whereas Prolific participants were compensated with USD 4. We excluded data from two university students who did not take the experiment seriously at all (i.e., reporting a 1 on a 6-point scale). One additional participant from Prolific was excluded for not following instructions during the autobiographical retrieval task (the person attempted to recall details of the lecture instead). This resulted in a total of 246 participants (*M*_age_ = 35.80, *N*_reported_ = 245) in all analyses. Participants were again randomly assigned to the four interpolated activities: 61 completed autobiographical retrieval, 68 semantic retrieval, 56 episodic retrieval, and 61 review. The sample size was slightly uneven across the conditions because of random assignment. See Table 2 for participant demographics.

#### 4.1.2. Materials and Procedure

The same materials and procedure were adopted from Experiment 1, except that all participants completed their experiment online.

### 4.2. Results

#### Only Content-Relevant Episodic Retrieval Enhanced New Learning

**Figure 2:**
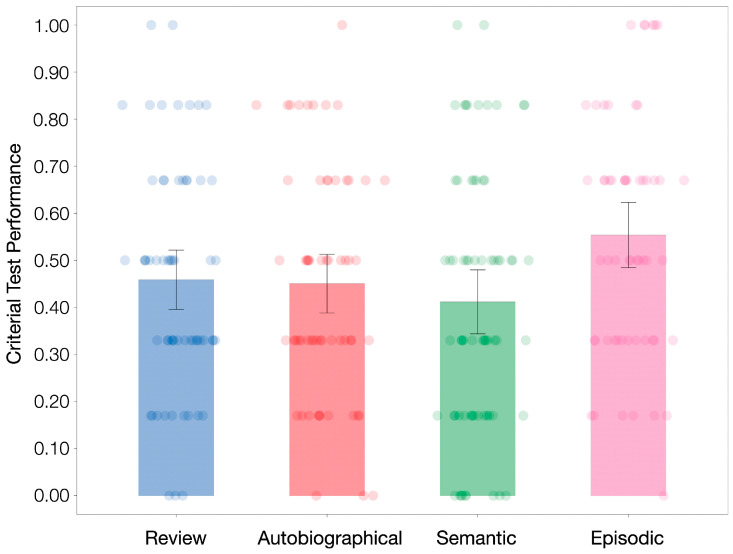
Criterial test performance across conditions of Experiment 2. Error bars denote 95% descriptive confidence interval. Direct comparisons reveal that episodic retrieval, but not semantic or autobiographical retrieval, significantly enhances new learning when compared to review.

Figure 2 shows the criterial test performance per condition. Similar to Experiment 1, an ANOVA showed a main effect of interpolated activity on criterial test performance, *F*(3, 242) = 3.24, *p* = 0.023, η_p_^2^ = 0.039. More importantly, only episodic retrieval (*M* = 0.55, *SD* = 0.26) enhanced new learning relative to review (*M* = 0.46, *SD* = 0.25), *t*(115) = 2.04, *p* = 0.044, *d* = 0.377. Once again, neither semantic retrieval (*M* = 0.41, *SD* = 0.28), *t*(127) = −1.00, *p* = 0.317, *d* = −0.177, nor autobiographical retrieval (*M* = 0.45, *SD* = 0.24), *t*(120) = −0.19, *p* = 0.854, *d* = −0.033, resulted in significantly better criterial test performance than review. If anything, both effects were in the negative direction. The data in the autobiographical retrieval condition are particularly noteworthy, given that participants in this condition showed marginally better criterial test performance than those in the review condition in Experiment 1. The complete absence of the effect here suggests that the marginal effect in Experiment 1 was perhaps unreliable. Similar to Experiment 1, lecture topics did not interact with interpolated activity, *F*(3, 238) = 0.19, *p* = 0.906, η_p_^2^ = 0.002. 

## 5. Exploratory Analyses

In the following, we report several exploratory analyses that used the combined data from Experiments 1 and 2 to increase statistical power. First, one might wonder if the inconclusive result of autobiographical retrieval on new learning in Experiment 1 was due to insufficient power. To address this concern, we report an exploratory analysis by combining the data from the two experiments. Next, we report a series of analyses aimed at quantifying context change and its contribution to criterial recall performance. We did not conduct these analyses using data from the individual experiments because they were not powered for these exploratory analyses.

### 5.1. Lecture Relevance as a Determinant of Test-Potentiated New Learning

An ANOVA with the interpolated activity and experiment (1 vs. 2) as independent variables and criterial test performance as the dependent variable showed a main effect for interpolated activity, *F*(3, 487) = 7.41, *p* < 0.001, η_p_^2^ = 0.044. Neither the main effect of the experiment, *F*(1, 487) = 2.64, *p* = 0.105, η_p_^2^ = 0.005, nor the interaction, *F*(3, 487) = 0.66, *p* = 0.580, η_p_^2^ = 0.004, was significant. Most importantly, similar to the conclusions from the individual experiments, interpolated episodic recall of content-relevant content promoted new learning, *t*(241) = 4.02, *p* < 0.001, *d* = 0.516, whereas autobiographical and semantic recall did not, *t*_autobiographical_(244) = 1.09, *p* = 0.275, *d* = 0.139; *t*_semantic_(252) = 0.41, *p* = 0.686, *d* = 0.051, despite the increase in statistical power of the combined data set. The null effect for interpolated autobiographical retrieval is particularly important, as the result in Experiment 1 was somewhat inconclusive. We now report data that bear relevance to the context change perspective.

### 5.2. Measuring Context Change and Its Impact on New Learning

As mentioned in the Introduction, we aimed to use interpolated task engagement as a measure of context change. If context change enhances new learning, one might expect a positive correlation between interpolated task engagement and criterial test performance. To ensure that any positive association between these measures was not simply driven by a general increase in effort, we factored out participants’ reports of seriousness in a hierarchical regression analysis. Specifically, the first model regressed seriousness on criterial test performance, and the second model regressed both seriousness and interpolated task engagement on criterial test performance. To quantify interpolated task engagement for autobiographical retrieval, we measured the total number of words that participants recalled during the two autobiographical retrieval trials. Two research assistants screened the autobiographical recall records and found that all participants were on-task (i.e., recalling what they were asked). To measure interpolated task engagement for semantic retrieval, we counted the number of correct exemplars recalled per participant based on Van Overschelde et al.’s (2004) category norms. To avoid double counting exemplars, we only included unique compound terms (e.g., Coke and Coca-Cola) but not repeated compound terms (e.g., beans, but not green beans and string beans).

We first report data for participants in the *episodic retrieval* condition. Here, we expect that interpolated recall would be positively associated with both criterial recall and seriousness (*M* = 4.99, *SD* = 1.00). Indeed, the regression model for seriousness was significant, *F*(1, 117) = 9.55, *p* = 0.002, *r* = 0.28. The second model, which included seriousness as well as recall probability of Segment 1 (*M* = 0.63, *SD* = 0.27) and Segment 2 (*M* = 0.63, *SD* = 0.30), was also significant, *F*(3, 115) = 11.22, *p* < 0.001, *r* = 0.48. Critically, the addition of Segments 1 and 2 recall probability significantly improve the model, *r*^2^_change_ = 0.15, *F*_change_ = 11.22, *p* < 0.001. Consequently, interpolated recall success predicted criterial recall performance beyond the contribution of overall task effort as measured by seriousness.

We now report data for participants in the other conditions. We first examine the data for participants in the *review* condition. Unlike participants in the episodic retrieval condition, seriousness (*M* = 4.98, *SD* = 1.01) was *not* significantly associated with criterial recall performance, *F*(1, 122) = 1.74, *p* = 0.189, *r* = 0.12. Because participants in the review condition did not complete any interpolated tasks that produced measurable data, we did not complete a hierarchical regression for them.

**Figure 3:**
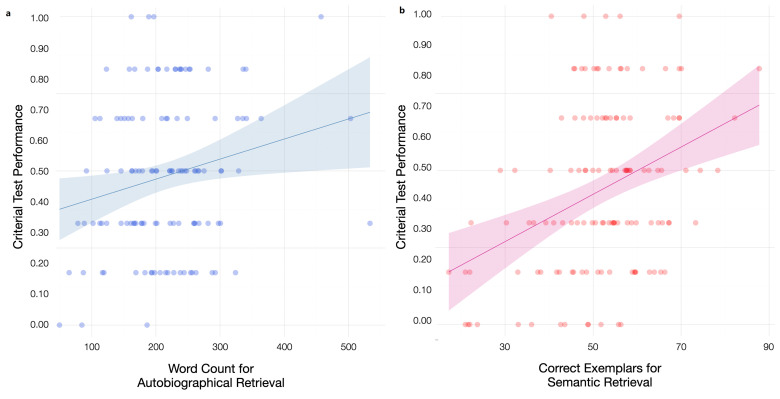
Relationship between engagement in content-irrelevant retrieval conditions and criterial test performance. Horizontal jitter is introduced to improve data visibility. We scale the amount of jitter to the content-irrelevant retrieval variable. The scatterplot in Panel (**a**) shows autobiographical retrieval engagement and criterial test performance. Engagement was measured by word count (horizontal jitter ±5). The scatterplot in Panel (**b**) shows semantic retrieval engagement and criterial test performance. Engagement is measured by the number of correct exemplars generated (horizontal jitter ±1).

For participants in the *autobiographical retrieval* condition, the first model showed that seriousness (*M* = 5.00, *SD* = 1.02) was weakly and positively associated with criterial test performance, *F*(1, 119) = 4.11, *p* = 0.045, *r* = 0.18. The second model, with the total word count of autobiographical retrieval as an additional predictor (*M* = 214.99 words, *SD* = 80.27 words), was also significant, *F*(2, 118) = 4.98, *p* = 0.008, *r* = 0.28. Most importantly, autobiographical recall significantly increased the predictive power of the regression model, *r*^2^_change_ = 0.04, *F*_change_ = 5.69, *p* = 0.019. Specifically, participants who were more engaged with the autobiographical recall task (as shown through a greater word count) showed better criterial test performance, and this association accounted for unique variance beyond overall task engagement as measured by seriousness. See Panel (a) of Figure 3 for a scatterplot.

Participants in the *semantic retrieval* condition exhibited a similar pattern to those in the autobiographical retrieval condition. Specifically, the first model revealed a marginal positive association between seriousness (*M* = 4.95, *SD* = 1.01) and criterial recall performance, *F*(1, 128) = 2.83, *p* = 0.095, *r* = 0.15. The second model, with the addition of the total number of correct exemplars generated across six categories (*M* = 51.92, *SD* = 13.07), was strongly predictive of criterial recall performance, *F*(2, 127) = 11.88, *p* < 0.001, *r* = 0.40. See Panel (b) of Figure 3 for a visual representation. Indeed, semantic retrieval success contributed substantially to the model, *r*^2^_change_ = 0.14, *F*_change_ = 20.49, *p* < 0.001. Together, the results from the semantic and autobiographical retrieval conditions show that, despite not conferring an overall benefit to new learning, participants who were more engaged with the interpolated tasks performed better on the criterial test. This outcome is consistent with the context change account.

## 6. Discussion

In two experiments, we consistently found that the interpolated episodic retrieval of lecture content promoted new learning of the lecture relative to the review. However, content-irrelevant interpolated retrieval, such as recalling a family vacation and childhood home or generating exemplars from category names, failed to promote new learning. This pair of experiments was the first to show that answering interpolated quiz questions can enhance learning of a video lecture in both laboratory and fully online settings. We now discuss their applied and theoretical implications.

### 6.1. Interpolated Retrieval Online and In-Lab

Although considerable research has demonstrated the benefits of interpolated quizzing on new learning (Chan et al., 2018a; Pastötter & Bäuml, 2014; Yang et al., 2018), most of this work employed experimental material that is unlikely to be encountered by students in the classroom. The contrived materials, such as word lists and paired associates, are advantages for theoretical investigations because they allow researchers to examine the data at a fine-grained level. However, they also limit the data’s generality. A few studies have demonstrated the benefits of interpolated retrieval on new learning with realistic lecture content, but, until very recently (Chan et al., 2025), all of these have tested participants in the laboratory, despite using the term “online lecture” to describe their material (e.g., Conrad & Newman, 2021; Pan et al., 2020; Szpunar et al., 2013). Here, Experiment 1’s participants were tested in the laboratory, and Experiment 2’s participants were tested online with the same materials and procedure, and we found that (i) answering interpolated quiz questions promoted new learning both in the laboratory (Experiment 1) and online (Experiment 2), and (ii) the benefits of interpolated retrieval appear smaller in Experiment 2 (*d* = 0.38) than in Experiment 1 (*d* = 0.64). Note that, when we compared the effect sizes in an ANOVA, the interaction between interpolated testing and the learning environment across the experiments was not significant, *F* = 1.00, *p* = 0.319. Nonetheless, our study was also not powered to detect this interaction.

Notably, the moderate interpolated retrieval benefit in our Experiment 2 mirrored that reported by Chan et al. (2025, *d* = 0.37, *N* = 235), who tested all of their participants online. Chan et al. (2025) suggested that the modest effect size in their experiment might be the result of online participants being distracted when attempting to learn the lecture material. In particular, they argued that typical in-lab studies present participants with a pristine learning environment, where they are not allowed to use mobile phones, browse the internet, talk to others, or engage with other distractions. The prohibition of these activities fosters a nearly ideal learning environment that diverges considerably from how students typically learn online (Adhani & Remijn, 2023; Aivaz & Teodorescu, 2022; Hollister et al., 2022). Consequently, the small FTE might reflect the level of benefits that one can expect from interpolated testing in distraction-filled online learning scenarios. Although this proposal is reasonable, Chan et al. (2025) did not test their lecture materials in the laboratory, so the small effect size could be a consequence of the materials rather than online learning being more distracting per se.

In the present study, the same lecture material produced non-significantly different effect sizes based on testing environments, so the lecture material is unlikely to be the culprit for the small (and nearly identical) effects exhibited in our Experiment 2 and in Chan et al. (2025). Rather, we tentatively conclude that online learning environments, which are often more distracting than in-lab studies, might diminish the benefits of interpolated testing. We emphasize that this hypothesis is tentative because it involves a cross-experimental comparison and different participant populations. Specifically, participants in Experiment 1 were university students, and participants in Experiment 2 featured both university students and Prolific participants. Even if we had enrolled only university students in Experiment 2, the participant population would still be confounded across experiments because those who self-select into in-person and online studies might have different characteristics. To fully remove this potential subject-selection confound, one must conduct a study in which participants are recruited for either an in-person or online study and are then randomly assigned to one of the environments after they have arrived at the laboratory. Specifically, participants cannot know where (in-lab or online) they would complete the experiment when they sign up or show up for the study. We are not aware of such a study at the current time, so a definitive answer to the question about online vs. in-person learning awaits further research. Moreover, we consider that Experiment 2 closely resembles the real-life learning contexts for online and asynchronous courses. At the very least, the present study showed that answering interpolated quiz questions, but not interpolated irrelevant retrieval activities, can promote the new learning of a video lecture both in-person and online.

### 6.2. Interpolated Retrieval, Strategy Change, and Context Change

In two experiments, we found that interpolated episodic retrieval enhanced subsequent learning, whereas interpolated semantic and autobiographical retrieval did not. The latter results have important theoretical and applied implications. Although researchers have sometimes reported that semantic recall can facilitate new learning (Divis & Benjamin, 2014; Pastötter et al., 2011), the effect was not always found (Weinstein et al., 2015). Recent studies have suggested that semantic generation potentiates new learning (Kliegl & Bäuml, 2021, 2023; Kriechbaum & Bäuml, 2024) only when the pre- and post-interpolated retrieval materials are unrelated to each other (e.g., when participants studied lists of noncategorized words), but not when they are related to each other (e.g., when participants studied inter-related word lists). Bäuml and colleagues explained the dissociation as supporting the context change account—specifically, performing semantic retrieval between the episodic encoding of word lists causes a mental context change that facilitates list discrimination. For example, after studying List 1, which is associated with a List 1 context, semantic generation alters the mental context from encoding to retrieval. When participants then study List 2, these words are encoded under a new, List 2 context. Later, when participants attempt to recall the words from only List 2, they can use the isolated List 2 context as a retrieval cue, thereby constraining retrieval candidates (Shimizu & Jacoby, 2005). This context change account assumes that, when participants do not perform retrieval between the noncategorized lists—such as by practicing mental arithmetic or restudy—the context between Lists 1 and 2 does not change. Therefore, when participants attempt to retrieve List 2 items later, a single study context is associated with both List 1 and 2 words, making it more difficult to achieve accurate recall and minimize intrusions (for evidence that reduced interference might not contribute to the forward testing effect, see Ahn & Chan, 2022; Boustani et al., 2023).

Why then would semantic generation fail to promote new learning for related items? According to Bäuml and colleagues, when the post-retrieval List 2 contains items from categories in a pre-retrieval List 1, the List 2 items remind participants of List 1 and reinstate the pre-retrieval context (Kliegl & Bäuml, 2021, 2023; Kriechbaum & Bäuml, 2024). Consequently, even if interpolated semantic generation changes participants’ mental context, the items in List 2 bring participants back to their List 1 context, thus making the retrieval of List 2 items as difficult as when participants did not perform an interpolated task that changes context (e.g., mental arithmetic, restudy). Critically, Bäuml and colleagues’ two-factor account attributes the benefits of episodic retrieval for the new learning of categorized items not to context change, but to strategy optimization. That is, when participants perform interpolated episodic retrieval, not only does it induce a mental context change, but the task also provides participants with valuable information about their own learning, and participants can use this metacognitive knowledge to optimize their subsequent encoding and retrieval (Ahn & Chan, 2024; Chan et al., 2020, 2018a). For example, participants might realize that they are underperforming their expectations and therefore exert greater time or effort into subsequent learning (Davis & Chan, 2023; Soderstrom & Bjork, 2014; Yang et al., 2017). Moreover, prior retrieval practice can promote memory organization during subsequent recall (Chan et al., 2020; Zaromb & Roediger, 2010).

In the present study, we found that neither semantic generation nor autobiographical recall promoted new learning of the lecture. This outcome is consistent with both the strategy-change account alone and the two-factor account. From the strategy-change perspective, content-irrelevant retrieval tasks should not help participants optimize their subsequent learning. From the two-factor account’s perspective, because the pre- and post-retrieval segments covered the same topic, the later segments might have reinstated the context of the prior segments and negated any context change benefits.

A key differentiator between these two accounts, however, is that we provided the first demonstration that greater context change, as quantified by interpolated task engagement, was associated with better criterial test performance, even when the overall task (i.e., autobiographical and semantic retrieval) did not produce a forward testing effect. This association is difficult to reconcile if one subscribes to the view that strategy change is the only contributor to the FTE, but it is consistent with the two-factor account.

Although the present results support the two-factor account, we continue to urge researchers to use caution when employing mental context change—which is unobservable—as an explanatory mechanism. We believe that the account of context change must satisfy two criteria for it to be truly useful: First, context change needs to be quantifiable independently of the criterial measure. Second, researchers should delineate why some tasks induce context change and others do not.

Regarding the first criterion of quantifying context change, one should not use better memory performance—which one is attempting to explain via context change—as a proxy for context change having occurred, because doing so creates a circular logic. The present study partly addressed this concern by providing a measure of context change during which participants engaged with the interpolated activity. But we emphasize that interpolated task engagement is only one possible way to assess context change, and our effort represents an early, preliminary attempt to measure it. In fact, even with these measurements, one cannot ascertain that a mental context change has occurred. Instead, other factors such as verbal fluency (which can affect both semantic generation and recall, Chan & McDermott, 2007b; Glisky et al., 1995) might underlie our measure of interpolated task engagement. Moreover, although Bauml and colleagues’ version of the context change account can explain why content-irrelevant retrieval tasks can promote new learning for unrelated materials but not related materials, this account requires one to accept *two* assumptions: first, that context change has occurred during the interpolated activity, and second, that related materials reinstate the pre-retrieval context. In this study, we sought to quantify the first assumption. Attempting to provide independent verification of the second assumption might prove even more difficult.

Concerning the second criterion, researchers should specify why some tasks (e.g., episodic and semantic retrieval) induce context change and others do not (e.g., mental arithmetic, drawing copy, restudy). Alternatively, researchers should provide careful conceptual analyses on the influence of a task on performance, without resorting to assumptions about whether a task causes context change. Some researchers have suggested that retrieval tasks (semantic, autobiographical, working memory, episodic) can induce context change relative to encoding, whereas tasks that do not involve retrieval (e.g., math) would not (Kliegl & Bäuml, 2021; Pastötter et al., 2011). We find this argument problematic because context change is meant to explain why retrieval potentiates new learning, so equating context change with retrieval again risks circularity. Further, this assumption fails to answer the question of why retrieval induces context change, whereas other tasks like math or drawing do not, even though these latter tasks seem just as different from episodic encoding. Indeed, in Tulving and Watkins’ (1974) interpolated drawing task, participants were shown a picture for 15 s and were then tasked to reproduce the picture by drawing it from short-term memory (similar to N-back). So it is unclear why this task would not enhance new learning from a context change perspective.

Bäuml and colleagues’ explanation for why semantic retrieval does not promote new learning for categorized material, however, might provide a promising way forward (Kliegl & Bäuml, 2021, 2023; Kriechbaum & Bäuml, 2024). Specifically, rather than focusing on whether or not a task would cause a context change, one might instead consider the contribution of multiple components of a task together. Applying this type of task analysis to the finding that interpolated mental arithmetic fails to promote new learning, the important question is perhaps not whether or why math does not cause a context change. Indeed, we believe that mental arithmetic would induce a context change from episodic encoding. Rather, it is possible that mental arithmetic is a difficult task for most participants, and they either experience minor mental fatigue from the task or develop disinterest in the task, which carries over to subsequent learning. If this were the case, one might expect that, unlike autobiographical recall (a self-relevant task) or semantic generation (an interesting and challenging task), greater task engagement for mental arithmetic would translate to poorer, instead of better, criterial test performance. To adequately test this idea, however, researchers need to control for participants’ math ability by giving those who are better at math more difficult questions. There are two advantages to this task analysis approach: First, researchers no longer need to explain why some tasks might induce a context change whereas other tasks might not. Second, one can produce testable hypotheses about the relationship between interpolated task engagement and new learning on a task-by-task basis.

Lastly, we would be remiss not to mention that not all empirical evidence has been consistent with the two-factor strategy and context account. For example, according to Bäuml and colleagues, interpolated non-episodic retrieval tasks should promote new learning when the pre- and post-retrieval items are not related to each other (Kliegl & Bäuml, 2021, 2023; Kriechbaum & Bäuml, 2024). However, Weinstein et al. (2015) showed that interpolated autobiographical and semantic retrieval failed to promote new learning, whereas episodic retrieval did, regardless of whether participants studied related or unrelated words across lists. Further research is needed to clarify support for the two-factor account.

### 6.3. Limitations and Constraints on Generality

In the present study, we defined content relevance as the extent to which the to-be-recalled content was related to the lecture material. Consequently, the episodic retrieval condition, in which participants were asked specific short-answer questions about the lecture, was considered content-relevant. In contrast, the autobiographical and semantic retrieval conditions were not content-relevant. However, we have not included an episodic retrieval task that asked participants short-answer questions unrelated to the just-lectured content. For example, imagine a student is attending a lecture on microbiology but has an exam on cognitive psychology during the next class period. The student might attempt to recall specific facts about cognitive psychology at various points during the microbiology lecture. Might such content-irrelevant retrieval enhance new learning of the microbiology lecture? Recent research suggests that the answer might be yes. For example, Yang et al. (2019) showed that the forward testing effect is transferable across different types of learning material. In one experiment, participants studied three successive sets of individual written statements about famous artists and then learned a set of painting-artist pairs. Following each of the three sets of written statements, participants either restudied the statements or retrieved them via fill-in-the-blank questions. Afterwards, participants learned a different type of material, which was a set of painting-artist pairs, and took a criterial test on it. A forward testing effect was found in this criterial test (see also Lee & Ahn, 2018). Therefore, recalling previously studied material can potentiate new learning of an unrelated set of new material.

When applying this logic to the example scenario above, it is reasonable to predict that recalling facts about cognitive psychology might facilitate the learning of microbiology. However, a critical difference between the Yang et al. (2019) finding and the content-irrelevant episodic retrieval example above is the following: In Yang et al., participants performed interpolated retrieval on the just-studied material. In contrast, in the content-irrelevant episodic retrieval example, the hypothetical student performs interpolated retrieval on other, distally studied material. However, because the forward testing effect transfers across domains and across a lag (Chan et al., 2018a; Kliegl & Bäuml, 2021), we tentatively predict that interpolated testing would enhance new learning even when the retrieved topic was not just-studied.

We also acknowledge the limitations in the generality of our findings, as we cannot extend them to longer lectures (e.g., 1–2 h) or semester-long learning. Future research is needed to test these possibilities. Further, should testing of a distal topic enhance new learning, it is important to determine the influence of topic proximity (in terms of the lag between initial learning and interpolated testing) on the magnitude of the forward testing benefit. Lastly, our data were collected with university students and participants from the Prolific online participant pool, and, although we included two lecture topics (statistics and physics), these materials do not resemble other types of materials that college students experience (e.g., group work, math, and art). Therefore, we urge caution when attempting to generalize these findings beyond the confines of our study.

## 7. Conclusions

The present experiments demonstrate that engaging in content-relevant episodic retrieval can reliably enhance new learning in both laboratory and online settings. Crucially, no benefits to new learning emerged from content-irrelevant autobiographical and semantic retrieval tasks among our stimuli and samples. These findings further show the robustness of the interpolated retrieval as an educational intervention, but they also highlight that retrieval alone is not sufficient to enhance new learning for some situations. Rather, it should be content-relevant. This outcome contrasts with previous findings that employed content-irrelevant retrieval among simplistic materials (Divis & Benjamin, 2014; Kliegl & Bäuml, 2021; Pastötter et al., 2011). Educators and students are encouraged to incorporate content-relevant episodic retrieval to promote learning. Educators may easily implement interpolated quizzes both during in-person lectures and online through short quizzes or clicker questions, requiring each student to engage in content-relevant episodic retrieval.

Our findings align with the idea that both strategy and context change contribute to the forward testing effect (Chan et al., 2018a; Kliegl & Bäuml, 2021; Yang et al., 2018). Engaging in episodic retrieval induces a context change and promotes strategy optimization, whereas autobiographical and semantic retrieval do not provide these same benefits. Consistent with Kliegl and Bäuml’s (2021) context change explanation, the reinstatement of the initial lecture context likely negates any context change benefits that content-irrelevant retrieval may offer. Additionally, the content-irrelevant retrieval tasks do not offer strategic benefits (e.g., potential gaps in knowledge, retrieval cues, and structure of criterion test), where episodic retrieval would. Thus, the two-factor account predicts that autobiographical and semantic retrieval would not produce the forward testing effect in instances where the learned materials are semantically related, as was the case in the presented study.

## Figures and Tables

**Table 1:** Experiment 1 participant demographic information.

Race	*N*
Asian/Asian American	20
Black/African American	7
Hispanic/Latinx	17
Multi-racial	2
Other	2
White	207
Decline to disclose	3
Gender	*N*
Man	80
Non-binary	2
Woman	166
Decline to disclose	1

*Note*. Participants were able to report multiple races.

**Table 2:** Experiment 2 participant demographic information.

Race	*N*
Asian/Asian American	21
Black/African American	50
Hispanic/Latinx	22
Multi-racial	7
Other	4
White	148
Decline to disclose	4
**Gender**	***N***
Agender	1
Man	121
Non-binary	4
Woman	115
Transgender	2
Decline to disclose	3

*Note*. Participants were able to report multiple races.

## Data Availability

The original data presented in this study are openly available in Open Science Framework (OSF) at https://osf.io/8mwpn/ (last updated on 7 March 2025).

## Abbreviations

The following abbreviations are used in this manuscript:

IRP	Interpolated retrieval practice
FTE	Forward testing effect
PI	Proactive interference
EEG	Electroencephalogram
ANOVA	Analysis of variance
